# Disabled-2 downregulation promotes epithelial-to-mesenchymal transition

**DOI:** 10.1038/sj.bjc.6605975

**Published:** 2010-11-09

**Authors:** J C Martin, B-S Herbert, B A Hocevar

**Affiliations:** 1 Department of Pharmacology and Toxicology, Indiana University School of Medicine, Indianapolis, IN 46202, USA; 2 Department of Medical and Molecular Genetics, Indiana University School of Medicine, Indianapolis, IN 46202, USA; 3 Department of Environmental Health, 1025 E. 7th Street, Room 189, Indiana University, Bloomington, IN 47405, USA

**Keywords:** disabled-2, EMT, TGF*β*, MAPK

## Abstract

**Background:**

Metastatic tumour cells are characterised by acquisition of migratory and invasive properties; properties shared by cells, which have undergone epithelial-to-mesenchymal transition (EMT). Disabled-2 (Dab2) is a putative tumour suppressor whose expression has been shown to be downregulated in various cancer types including breast cancer; however, its exact function in suppressing tumour initiation or progression is unclear.

**Methods:**

Disabled-2 isoform expression was determined by RT–PCR analysis in human normal and breast tumour samples. Using shRNA-mediated technology, Dab2 was stably downregulated in two cell model systems representing nontumourigenic human mammary epithelial cells. These cells were characterised for expression of EMT markers by RT–PCR and western blot analysis.

**Results:**

Decreased expression of the p96 and p67 isoforms of Dab2 is observed in human breast tumour samples in comparison to normal human breast tissue. Decreased Dab2 expression in normal mammary epithelial cells leads to the appearance of a constitutive EMT phenotype. Disabled-2 downregulation leads to increased Ras/MAPK signalling, which facilitates the establishment of an autocrine transforming growth factor *β* (TGF*β)* signalling loop, concomitant with increased expression of the TGF*β*2 isoform.

**Conclusion:**

Loss of Dab2 expression, commonly observed in breast cancer, may facilitate TGF*β*-stimulated EMT, and therefore increase the propensity for metastasis.

Breast cancer remains the most prevalent form of cancer diagnosed and the second-leading cause of cancer deaths in women. The ultimate cause of death in breast cancer is generally not because of the primary tumour itself, but rather from metastatic spread of the tumour. Metastasis is a coordinated process, which involves intravasation of tumour cells from the primary site into the circulation, followed by extravasation and establishment of a secondary tumour in a target organ (Hunter *et al*, 2008). Tumour cells that possess metastatic capability have been shown to acquire fibroblastoid invasive properties, which allows for subsequent degradation and migration through the extracellular matrix. These same properties are characteristic of cells, which have undergone epithelial-to-mesenchymal transition (EMT; Berx *et al*, 2007; Yang and Weinberg, 2008).

Epithelial-to-mesenchymal transition is defined as the loss of epithelial characteristics and the acquisition of a mesenchymal phenotype (Thiery and Sleeman, 2006). Concurrently, cells undergoing EMT alter gene expression patterns from genes required to maintain epithelial morphology, such as E-cadherin, to expression of mesenchymal genes, such as N-cadherin, vimentin and fibronectin. Although cooperation between several signalling pathways occurs during EMT, signalling by the transforming growth factor *β* (TGF*β*) cytokine family has emerged as a key inducer of EMT in both developmental and pathological settings (Xu *et al*, 2009). Transforming growth factor *β* functions as a potent tumour suppressor in normal epithelial cells by inhibition of cell proliferation, maintenance of genomic stability, and stimulation of cell differentiation and apoptosis (Massague and Gomis, 2006). An unexpected role for TGF*β* as a pro-metastatic factor, however, has been shown to occur late in tumour progression (Tang *et al*, 2003), which may be attributed to the ability of TGF*β* to stimulate EMT. Recent studies have demonstrated that human mammary epithelial cells, which have undergone EMT, acquire stem cell-like characteristics, become invasive and exhibit resistance to chemotherapy, which could also be recapitulated in cultured cells by treatment with TGF*β* (Mani *et al*, 2008). In addition, autocrine production of TGF*β*, stimulated by activation of the Ras/Raf/MAPK pathway, has been shown to stabilise the EMT phenotype *in vitro* and *in vivo* (Lehmann *et al*, 2000; Janda *et al*, 2002).

Disabled-2 (Dab2) or deleted in ovarian carcinoma-2 is a putative tumour suppressor first identified in a screen for transcripts downregulated in ovarian tumours *vs* normal ovarian surface epithelial cells (Mok *et al*, 1994). Disabled-2 has subsequently been shown to be downregulated in a variety of human tumour types including prostate (Tseng *et al*, 1998), bladder (Karam *et al*, 2007), oesophageal (Anupam *et al*, 2006), colorectal (Kleeff *et al*, 2002) and metastatic pancreatic cancer (Huang *et al*, 2001). Comparison of genes differentially expressed in an *in vivo* mouse mammary carcinogenesis model indicated that Dab2 was downregulated in 80% of mammary tumours (Schwahn and Medina, 1998). In human breast tumours, loss of Dab2 protein expression was observed in 74% of samples analyzed, whereas expression in 10 normal breast tissue samples was maintained (Bagadi *et al*, 2006). These results suggest that Dab2 may function as a tumour suppressor in breast cancer; however, the exact role of Dab2 in prevention of tumour initiation or progression is unclear. Disabled-2 has been shown to have a variety of diverse roles within the cell. Disabled-2 can facilitate endocytosis through its association with clathrin, the clathrin adaptor protein AP-2, and myosin VI (Morris and Cooper, 2001; Morris *et al*, 2002). In addition Dab2, through its N-terminal PTB domain, can directly bind to cell surface receptors such as members of the LDL receptor family and various integrin subunits (Morris and Cooper, 2001; Gallagher *et al*, 2004). Last, Dab2 has been shown to modulate various signalling pathways, including the TGF*β* (Hocevar *et al*, 2001), Wnt (Hocevar *et al*, 2003), JNK (Hocevar *et al*, 2005), Ras/MAPK (Xu *et al*, 1998; Zhou and Hsieh, 2001) and Src pathways (Zhou *et al*, 2003).

Cross-talk between the TGF*β* and Ras-MAPK signalling pathways has been postulated to be required for both induction and maintenance of EMT (Janda *et al*, 2002). As Dab2 has been shown to modulate both pathways, we have explored whether loss of Dab2 expression in nontumourigenic human mammary epithelial cells affects TGF*β*-stimulated EMT. We show here that downregulation of Dab2 expression leads to activation of Ras/MAPK signalling, increased production of TGF*β*, and the acquisition of a stable EMT phenotype.

## Materials and Methods

### Cell culture, treatment and establishment of stable cell lines

MCF10A1 cells were obtained from the Barbara Ann Karmanos Cancer Center (Detroit, MI, USA) and cultured in DMEM/F12 media (Mediatech, Manassas, VA, USA) containing 5% equine serum (Invitrogen, Carlsbad, CA, USA), 20 ng ml^−1^ EGF, 10 *μ*g ml^−1^ insulin, 0.5 *μ*g ml^−1^ hydrocortisone, 100 ng ml^−1^ cholera toxin (all purchased from Sigma-Aldrich, St Louis, MO, USA) and 1 × antibiotic/antimycotic solution (Gemini Bioproducts, Sacramento, CA, USA). HME5-cdk4 cells (Ramirez *et al*, 2003) were cultured in Medium 171 (Cascade Biologics, Portland, OR, USA) supplemented with 50 mg ml^−1^ bovine pituitary extract (Invitrogen), 20 ng ml^−1^ EGF, 10 *μ*g ml^−1^ insulin, 0.5 *μ*g ml^−1^ hydrocortisone and 1 × antibiotic/antimycotic solution. Recombinant human TGF*β*1 was obtained from R&D Systems (Minneapolis, MN, USA) and reconstituted as suggested. SB431542 was purchased from Sigma and U0126 was purchased from Calbiochem (San Diego, CA, USA).

Nucleotide sequences were chosen for stable ablation of human Dab2 using shRNA-mediated technology beginning at nucleotides 600 and 1060 (sh599-F 5′-GCAACAGGCTGAACCATTA-3′ sh1059-F 5′-TCGACACCTTCTTCGTTTG-3′), based on Genbank accession U53446. DNA oligos were synthesised (Invitrogen), annealed and inserted into pSIREN-retroQ (Clontech, Mountain View, CA, USA) according to manufacturer's instructions. Following sequence validation (DNA Sequencing Core Facility, Indiana University), negative control (provided by Clontech) and shDab2 constructs were cotransfected along with pCL-10A1 (Imgenex, San Diego, CA, USA) into 293T packaging cells. Retrovirus was harvested from the media after 48 h and used to infect parental cells in the presence of 8 *μ*g ml^−1^ polybrene (Sigma-Aldrich), followed by selection in media containing 1 *μ*g ml^−1^ puromycin (Gemini Bioproducts). Stable pools of cells expressing the various constructs were maintained in their respective media with puromycin.

### Cell lysis, immunoprecipitation and western blot analysis

Whole cell extracts were prepared by lysis in buffer D (20 mM Tris, pH 7.5, 1%, Triton X-100, 10% glycerol, 137 mM NaCl, 2 mM EDTA, 25 mM *β*-glycerophosphate, 20 mM Na_3_VO_4_ and COMPLETE EDTA-free protease inhibitor mixture; Roche Diagnostics, Indianapolis, IN, USA), sonicated and protein concentrations were measured using Bio-Rad Protein Assay Dye Reagent Concentrate (Bio-Rad Technologies, Hercules, CA, USA). Equal amounts of protein were resolved on SDS–PAGE gels, transferred to PVDF membranes (Immobilon, Millipore, Billerica, MA, USA) and subjected to western blot analysis. For immunoprecipitation, lysates were pre-cleared with protein G-Sepharose (Zymed, San Francisco, CA, USA), after which primary antibody (1 *μ*g) was added. After 4 h, protein G-Sepharose beads were added for an additional 2 h. Following five washes with modified RIPA buffer (50 mM Tris pH 7.4, 150 mM NaCl, 1% NP-40, 0.25% deoxycholate, 1 mM EDTA), immunoprecipitated proteins were eluted with 2 × Laemmli sample buffer and subjected to SDS–PAGE and western blot analysis. Blots were developed using enhanced chemiluminescence (ECL-Plus, GE Healthcare, Pittsburgh PA, USA) and visualised by autoradiography. The following antibodies were used for western blot analysis and immunoprecipitation: Dab-2/p96 (610465) and N-cadherin (610921) from BD Transduction Laboratories (San Jose, CA, USA); p-ERK (sc-7383), ERK2 (sc-154), Grb2 (sc-255), HSP90 *α* (sc-7947), Sos (sc-17793) and Vimentin (sc-5565) from Santa Cruz Biotechnology (Santa Cruz, CA, USA).

### Quantitative RT–PCR analysis and Origene PCR array (Origene; Rockville, MD, USA)

Total RNA was purified using the Qiagen RNeasy Mini Prep Kit according to manufacturer's instructions. Using 1.0–2.5 *μ*g of total RNA, cDNA was synthesised with SuperScript II RT (Invitrogen), diluted and qPCR was performed for the indicated genes in triplicate using gene specific primers and SYBR Green detection (Roche Diagnostics) on an Applied Biosystems 7900 HT Fast System using the following cycling parameters: 95°C for 10 min, followed by 40 cycles of 95°C for 15 s and 60°C for 1 min. After normalisation to *β-*actin, relative mRNA levels were quantitated by the ΔΔCt method. Transcript quantitation levels are expressed relative to that observed in untreated cells, which was set to 1.0. Shown is the mean±s.d. from an individual experiment; individual experiments were conducted a minimum of three times. Primer sequences used for qPCR analysis are provided in Supplementary Data (Supplementary Table 2).

Determination of p96 and p67 isoform expression levels in normal and breast tumour samples was performed utilising the Human Breast Cancer Rapid-Scan Panel (Origene) according to manufacturer's suggestions and performed in duplicate using the two individual plates provided. Disabled-2 PCR was performed on cDNA samples arrayed at 1000 × concentration, whereas actin PCR was performed on samples arrayed at 1 × concentration using primers provided by the manufacturer. Primers for Dab2 isoform discrimination were as follows: forward 5′-GGGAGTGAGGCCCTAATGATT-3′ and reverse 5′-GTTCTGAGACGGGAGGAGCAAAG-3′. Reactions were analyzed by gel electrophoresis in 1% agarose gels containing ethidium bromide and visualised by UV transillumination. Images were captured using the Kodak EDAS 290 Electrophoresis Documentation and Analysis System (Kodak; Rochester, NY, USA).

## Results

### Disabled-2 isoform expression is decreased in human breast cancer

**Figure 1:**
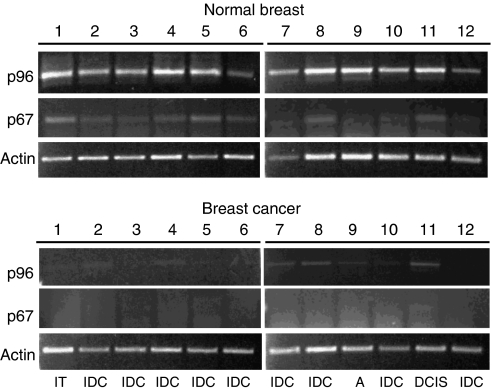
Human breast tumours exhibit decreased expression of the p96 and p67 forms of Dab2. Amplified cDNA prepared from 12 normal and 12 breast tumour samples arrayed in a multi-well PCR plate was obtained from the manufacturer (Origene). PCR analysis was carried out with primers flanking the splice site for generation of the p67 isoform of Dab2 and primers for human actin as control. Products were resolved on a 1% agarose gel and visualised by ethidium bromide staining. Amplification of the p96 isoform results in a product of 822 bp, whereas amplification of the p67 isoform yields a product of 167 bp. Images were obtained using the Kodak 1D programme following ultraviolet illumination. Histopathological data for individual BC samples is detailed in Supplementary Data. Abbreviations: A=adenoid cystic; DCIS=ductal carcinoma *in-situ*, IDC=invasive ductal carcinoma; IT=invasive mixed tubular.

Disabled-2 is expressed as two major cellular isoforms, the full-length p96 and the p67 isoforms, which are generated from the same transcript by alternative splicing (Xu *et al*, 1995). Decreased Dab2 protein expression has been shown in human breast tumours by immunohistochemistry; however, the antibody utilised does not discriminate between the two isoforms (Bagadi *et al*, 2006). To further extend these observations, we probed the relative mRNA abundance of the p96 and p67 isoforms of Dab2 in 12 normal human breast tissue and 12 breast cancer samples using the Human Breast Cancer Rapid-Scan Panel (Origene). By utilising PCR primers, which flank the splice site in p67, the relative abundance of the two isoforms can be determined based on differing sizes of the amplified products. As shown in Figure 1, normal breast tissue, obtained from patients undergoing reduction mammoplasty, expresses predominantly the p96 isoform; in addition, expression of the p67 isoform can be detected in the majority of samples. In contrast, samples derived from breast tumours demonstrate a marked reduction in expression of the p96 isoform, whereas the p67 isoform is virtually undetectable (Figure 1). Of note is the presence, but decreased expression of detectable p96 and p67 isoforms in the ductal carcinoma *in situ* (DCIS) sample (No. 11). The presence or absence of Dab2 mRNA expression does not appear to correlate with levels of expression of either the oestrogen or progesterone receptors as determined by the manufacturer (Supplementary Table 1). Although we cannot ascertain that the normal mammary tissue utilised in this study contains equal amounts of epithelial cells, the data presented here suggest that downregulation of both the p96 and p67 isoforms of Dab2 occurs frequently during breast cancer progression.

### Downregulation of Dab2 leads to appearance of a stable EMT phenotype

**Figure 2:**
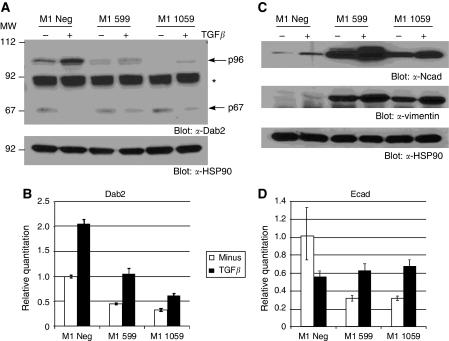
Downregulation of Dab2 leads to constitutive EMT. (**A**) Cell lysates were prepared from untreated or cells treated with 2.5 ng ml^−1^ TGF*β* for 48 h followed by western blot analysis for Dab2 expression as described in Materials and Methods. Equal protein loading is demonstrated by western blot analysis for HSP90. Arrows indicate the position of the p96 and p67 Dab2 isoforms; Asterisk indicates a non-specific band. (**B**, **D**) Detection of Dab2 or E-cadherin by qRT–PCR analysis in untreated and cells treated with 2.5 ng ml^−1^ TGF*β* for 48 h was performed as described in Materials and Methods. Quantitation is expressed relative to levels in untreated M1 Neg cells, which is set to 1.0. Shown is the mean expression value ±s.d. from an individual experiment; *n*=3 independent experiments. (**C**) Upregulation of EMT marker proteins following knockdown of Dab2. Cell lysates from unstimulated or cells treated with 2.5 ng ml^−1^ TGF*β* for 48 h were subjected to western blot analysis for N-cadherin (Ncad) and vimentin expression as described in Materials and Methods. HSP90 expression by western blot analysis demonstrates equal protein loading (*n*=3 independent experiments).

To examine the role of Dab2 as a tumour suppressor in breast cancer, we chose to stably downregulate Dab2 expression utilising shRNA-mediated technology in the normal human mammary epithelial cell line MCF10A1, which harbours a defective p16/INK4a locus (Tang *et al*, 2003, 2007) allowing for continued cell growth. To determine the effectiveness of the stable knockdown, we examined both basal and TGF*β*-stimulated levels of Dab2, as TGF*β* has previously been shown to upregulate Dab2 expression (Jechlinger *et al*, 2003). Western blot analysis of lysates from cells expressing the control virus, designated M1 Neg, show upregulation of the p96 isoform following 48 h treatment with TGF*β* (Figure 2A). In contrast, M1 cells expressing the sh-599 and sh-1059 targeted constructs show reduced basal as well as TGF*β*-stimulated induction of the p96 Dab2 isoform (Figure 2A). The p67 isoform expression is shown to be reduced following TGF*β* treatment in all cell lines, whereas basal levels are unaffected by the shRNA targeting constructs. To confirm that Dab2 is downregulated at the mRNA level, we performed qRT–PCR analysis on the various cell lines. As shown in Figure 2B, TGF*β* treatment of M1 Neg cells leads to two-fold upregulation of Dab2 message; in contrast, reduced basal and TGF*β*-stimulated Dab2 levels are observed in cell lines expressing the sh-599 and sh-1059 constructs.

During establishment of stable Dab2 knockdown pools, we did not observe significant effects of Dab2 ablation on cell proliferation; however, we did observe differences in cellular morphology between these cells and cells expressing only the negative control virus (Supplementary Figures 1 and 2). Untreated M1 Neg cells exhibit a cuboidal morphology, characteristic of a normal epithelial phenotype; however, following treatment with TGF*β*, the cells acquire a fibroblastic appearance, characteristic of cells, which have undergone EMT (Supplementary Figure 1). In contrast, untreated M1 599 and M1 1059 cells already exhibit a mesenchymal morphology, which can be further accentuated on treatment with TGF*β* (Supplementary Figure 1). During EMT, while expression of E-cadherin is lost, expression of N-cadherin and vimentin is gained. To confirm that the observed morphological changes are consistent with EMT, we examined the expression of N-cadherin and vimentin by western blot analysis and E-cadherin mRNA expression by qRT–PCR analysis. Although TGF*β* treatment of M1 Neg cells causes increased expression of N-cadherin and vimentin, large increases in basal as well as TGF*β*-stimulated expression of both proteins are observed in cells expressing both the sh-599 and sh-1059 constructs (Figure 2C). In addition, increased mRNA expression of N-cadherin and vimentin is observed in Dab2 knockdown cells in comparison to parental M1 Neg cells, which is maintained under both sparse and confluent cell culture conditions (Supplementary Figure 3). At the mRNA level, M1 Neg cells treated with TGF*β* exhibit a 50% reduction in E-cadherin mRNA levels, whereas basal mRNA levels for E-cadherin are reduced in M1 599 and M1 1059 cells by ∼70% (Figure 2D). Decreased expression and relocalisation of E-cadherin to the cytosol was also observed in M1 Neg cells treated with TGF*β,* although untreated M1 599 and M1 1059 cells exhibited these same changes as determined by immunoflourescence microscopy (Supplementary Figure 1).

### Loss of Dab2 expression leads to activation of the Ras/MAPK signalling pathway and establishment of an autocrine TGF*β* signalling loop

**Figure 3:**
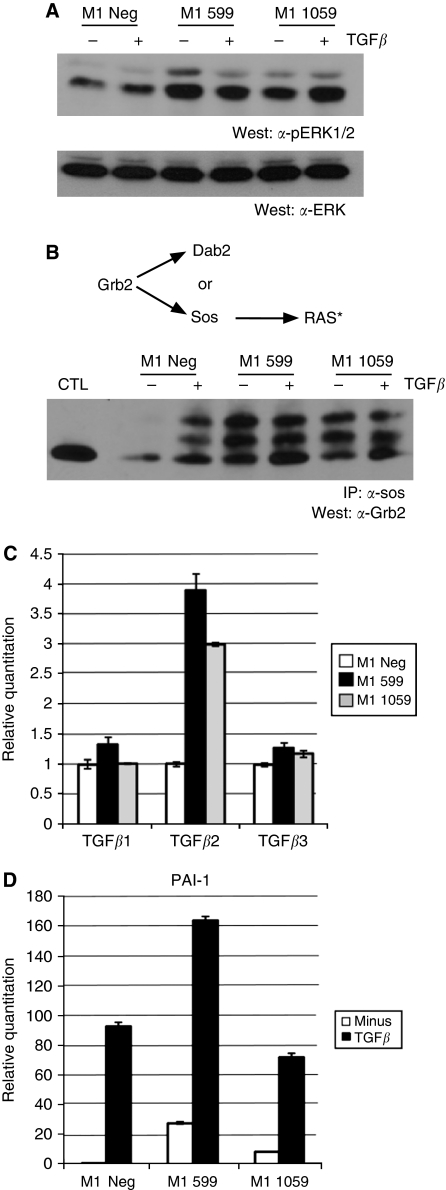
Activation of Ras/MAPK signalling in Dab2 knockdown cells leads to establishment of an autocrine TGF*β* signalling loop. (**A**) M1 Neg, M1 599 and M1 1059 cells were unstimulated or stimulated with TGF*β* (2.5 ng ml^−1^) for 48 h and subjected to western blot analysis for phospho-ERK1/2 (pERK1/2) and total ERK2 expression as described in Materials and Methods. (**B**) Equal amounts of cell lysates from TGF*β*-treated or untreated cells were immunoprecipitated with *α*-Sos antibodies followed by western blot analysis for Grb2 as described in Materials and Methods. Cell lysate from untreated M1 Neg cells was run as a control (CTL) for Grb2 visualisation. Asterisk denotes activated RAS (**C**, **D**) TGF*β*1, TGF*β*2 and TGF*β*3 mRNA levels were determined by qRT–PCR analysis in untreated (**C**) cells, whereas PAI-1 mRNA (**D**) was determined in untreated cells and in cells treated with 2.5 ng ml^−1^ TGF*β* as described in Materials and Methods. Expression is shown relative to levels in untreated M1 Neg cells, which is set to 1.0. Shown is the mean expression value ±s.d. from an individual experiment; *n*=3 independent experiments.

Previous studies have demonstrated that Dab2 overexpression can suppress activation of the MAPK pathway (Zhou and Hsieh, 2001). As Ras/MAPK signalling has been shown to cooperate with TGF*β* signalling to elicit EMT (Janda *et al*, 2002), we next investigated whether MAPK signalling in mammary epithelial cells is altered by suppression of Dab2 expression. Activation of Ras initiates a signalling cascade involving activation of Raf, which leads to the phosphorylation and activation of MEK1/2, and subsequent phosphorylation and activation of ERK1/2. As determined by western blot analysis, we observed markedly increased levels of phosphorylated ERK1 and ERK2 (p-ERK1/2) in M1 599 and M1 1059 cells, as compared with levels in M1 Neg cells (Figure 3A). The C-terminal proline-rich domain of Dab2 has been shown to bind to the adaptor molecule Grb2 and prevent its association with Sos, thereby decreasing activation of the Ras signaling cascade (Xu *et al*, 1998; Zhou and Hsieh, 2001). We next assessed whether loss of Dab2 expression drives activation of the Ras pathway leading to increased p-ERK1/2 by causing increased association of Grb2 with Sos. To determine this, cell lysates from M1 Neg, M1 599 and M1 1059 cells stimulated with TGF*β* for 48 h were subjected to immunoprecipitation with *α*-Sos antibody followed by western blot analysis for Grb2. As seen in Figure 3B, although TGF*β* treatment leads to increased Grb2/Sos association in M1 Neg cells, the level of Grb2/Sos association is significantly enhanced in the Dab2 knockdown cell lines. In addition, we observed the appearance of slower migrating forms of Grb2 in the TGF*β*-treated M1 Neg and basal as well as TGF*β*-treated immunoprecipitates from M1 599 and M1 1059 cells, which has been attributed to tyrosine phosphorylation of Grb2 (Li *et al*, 2001).

Activation of MAPK activity has been postulated to synergise with TGF*β* in induction of EMT (Janda *et al*, 2002), whereas maintenance of EMT through establishment of an autocrine TGF*β* signalling loop has been shown to require high levels of MAPK activity (Lehmann *et al*, 2000). As many of the changes in morphological appearance and gene expression patterns in Dab2 knockdown cells recapitulate those observed following treatment of control cells with TGF*β*, we examined whether these cells exhibit increased mRNA expression of the three mammalian TGF*β* isoforms, TGF*β*1, TGF*β*2 and TGF*β*3. As shown in Figure 3C, although TGF*β*1 and TGF*β*3 levels are modestly elevated in M1 599 and M1 1059 cells in comparison to M1 Neg cells, TGF*β*2 levels are increased three- to four-fold in Dab2 knockdown cells. To assess whether the increased mRNA levels of the TGF*β* isoforms leads to increased production of active TGF*β* and enhanced TGF*β* signalling, we examined the mRNA expression of *PAI-1*, a classical TGF*β*-stimulated gene (Piek *et al*, 2001). As seen in Figure 3D, stimulation of M1 Neg cells with TGF*β* leads to upregulation of PAI-1; however, in M1 599 and M1 1059 cells basal expression levels of PAI-1 are significantly elevated, which can be further augmented by exogenous TGF*β* treatment. These results thus suggest that the increased MAPK signalling exhibited in Dab2 knockdown cells leads to establishment of an autocrine TGF*β* signalling loop.

### Blockade of Ras/MAPK or TGF*β* signalling partially reverts the constitutive EMT phenotype of Dab2 knockdown cells

**Figure 4:**
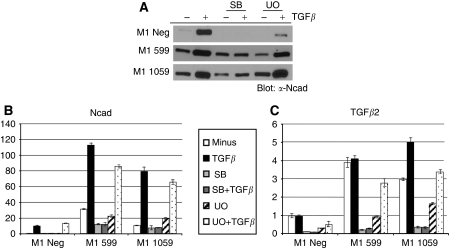
Inhibition of TGF*β* or MAPK signalling abrogates TGF*β*-mediated EMT. (**A**) M1 Neg, M1 599 and M1 1059 cells were either untreated or pre-treated for 30 min with 10 *μ*M SB431542 (SB) or 10 *μ*M U0126 (UO) followed by addition of TGF*β* (2.5 ng ml^−1^) for 48 h. Cell lysates were prepared and western blot analysis performed to determine N-cadherin (Ncad) expression levels as described in Materials and Methods (*n*=2 independent experiments). (**B**, **C**) qRT–PCR analysis was performed to determine relative expression levels of N-cadherin, TGF*β*1, TGF*β*2 and TGF*β*3 mRNA following the treatments described in **A**. Shown is the mean±s.d. of triplicate assays with M1 Neg unstimulated levels set to 1.0 (*n*=2 independent experiments).

As we have observed both upregulation of Ras/MAPK signalling and increased expression of TGF*β* isoforms in Dab2 knockdown cells, we next asked whether blockade of either pathway using pharmacological inhibitors reverts the constitutive EMT phenotype observed. To assess this, M1 Neg, M1 599 and M1 1059 cells were treated with a specific T*β*RI receptor kinase inhibitor (SB431542) or a MEK1 inhibitor (U0126), in the presence or absence of exogenous TGF*β* for 48 h. Treatment of M1 Neg cells with SB431542 led to loss of TGF*β*-stimulated induction of N-cadherin mRNA and protein expression, whereas treatment with U0126 led to a significant decrease in TGF*β*-mediated induction of protein expression only (Figure 4A and B). Similarly, M1 599 and M1 1059 cells treated with SB431542 failed to induce N-cadherin mRNA and protein following TGF*β* treatment; however, SB431542 treatment also led to a significant reduction in basal mRNA levels for N-cadherin. In contrast, although U0126 treatment decreased basal N-cadherin levels in M1 599 cells only, TGF*β*-stimulated induction of mRNA and protein expression remained comparable (Figure 4A and B). To determine whether the decreased basal expression of N-cadherin observed after treatment with SB431542 and U0126 correlates with a blockade of autocrine TGF*β* signalling, we examined the mRNA expression of the TGF*β*2 isoform, which exhibits the largest increase in expression. We find that both SB431542 and to a lesser extent U0126 treatment leads to a significant decrease in basal expression of TGF*β*2 in M1 Neg, M1 599 and M1 1059 cells.

### Downregulation of Dab2 in HME-cdk4 cells facilitates TGF*β*-mediated EMT

**Figure 5:**
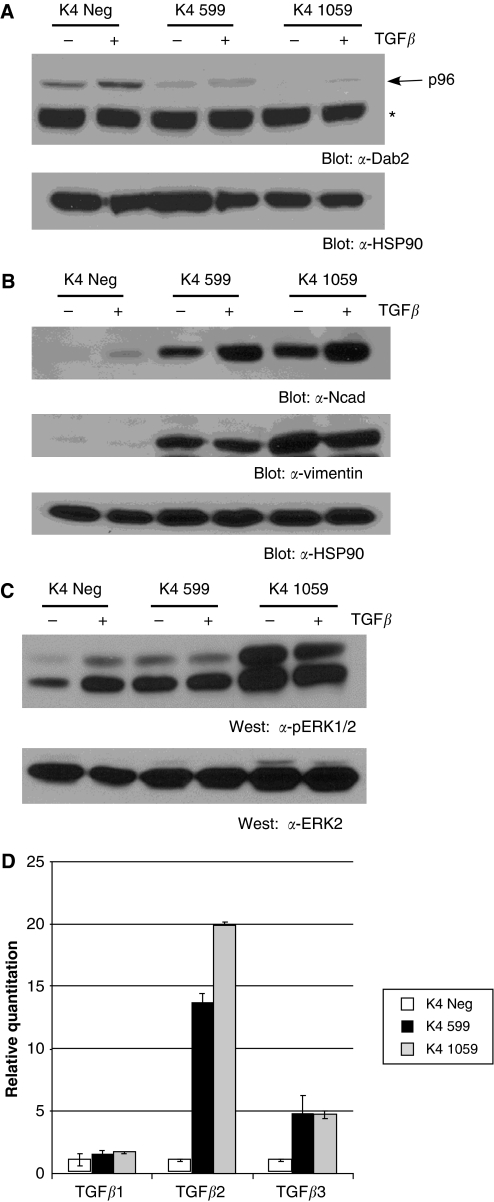
Downregulation of Dab2 in HME5-cdk4 (K4) cells results in a constitutive EMT phenotype. (**A**, **B**) Cell lysates from unstimulated cells or cells treated with 2.5 ng ml^−1^ TGF*β* for 48 h were subjected to western blot analysis for Dab2, N-cadherin (Ncad) and vimentin expression as described in Materials and Methods. HSP90 expression by western blot analysis demonstrates equal protein loading (*n*=3 independent experiments). Arrow indicates the position of the p96 Dab2 isoform; Asterisk indicates a non-specific band. (**C**) K4 Neg, K4 599 and K4 1059 cells were untreated or stimulated with TGF*β* (2.5 ng ml^−1^) for 48 h and subjected to western blot analysis for phospho-ERK1/2 (pERK1/2) and total ERK2 expression as described in Materials and Methods. (**D**) Elevated expression levels of TGF*β* isoforms exhibited by Dab2 knockdown cells. Transforming growth factor *β*1, TGF*β*2 and TGF*β*3 mRNA expression was determined by qRT–PCR analysis in untreated K4 Neg and shDab2-expressing cells as described in Materials and Methods. Shown is mean±s.d. from assays performed in triplicate with expression in K4 Neg set to 1.0. (*n*=3 independent experiments).

To extend these studies to another nontumourigenic mammary epithelial cell line, we performed similar experiments in the HME5-cdk4 (K4) cell line, derived from a patient undergoing breast augmentation, which possesses an extended *in vitro* culture lifespan due to overexpression of cdk4 (Ramirez *et al*, 2003). As shown in Figure 5, stable knockdown of Dab2 in HME-cdk4 cells (Figure 5A) leads to a constitutive EMT phenotype, characterised by increased protein expression of N-cadherin and vimentin (Figure 5B). In addition, similar to results obtained in M1 599 and M1 1059 cells, elevated Ras/MAPK signalling and increased expression of TGF*β*2 and TGF*β*3 is observed in the Dab2 knockdown cell lines (Figure 5C and D).

## Discussion

### Loss of Dab2 expression is associated with breast cancer progression and EMT

**Figure 6:**
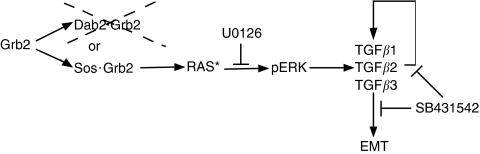
Loss of Dab2 facilitates EMT. In the basal state, cytosolic Grb2 can be found in association with Dab2 or Sos. Loss of Dab2 expression, commonly observed in breast cancer, is postulated to increase the proportion of Grb2 associated with Sos, resulting in Ras activation (RAS^*^), stimulation of MAPK signalling and phosphorylation of ERK (pERK). Activation of ERK stimulates increased transcription of the TGF*β* isoforms, in particular the TGF*β*2 isoform. Increased TGF*β* stimulation induces transient EMT, and also initiates establishment of an autocrine TGF*β* signalling loop leading to a stable EMT phenotype exemplified by increased expression of EMT markers. Inhibition of TGF*β* signalling with SB431542 or MAPK signalling with U0126 decreases TGF*β* production and causes partial reversion of the EMT phenotype.

The process of epithelial-to-mesenchymal transition, although critical for proper embryonic development, has recently been linked with aggressive, invasive and metastatic behaviour of various cancer types, including breast cancer (Yang and Weinberg, 2008; Tan *et al*, 2009). Transforming growth factor *β* signalling exerts potent growth–inhibitory effects in normal epithelial cells; however, late in tumour progression, TGF*β* has been shown to cooperate with other pro-oncogenic pathways to support tumour progression, which may be due to the ability of TGF*β* to stimulate EMT (Xu *et al*, 2009). Indeed, increased levels of TGF*β* have been observed in human breast cancer samples, which are associated with expression of a pro-invasive, angiogenic and metastatic gene expression profile (Sarrio *et al*, 2008). Here, we demonstrate that decreased expression of the tumour suppressor Dab2 leads to activation of Ras/MAPK signalling, which initiates the establishment of an autocrine TGF*β* signalling loop and stable EMT (Figure 6).

Although decreased Dab2 mRNA levels were observed in all human breast cancer samples analyzed, irrespective of tumour grade, the decreased expression of Dab2 in the DCIS sample suggests that Dab2 loss occurs early in breast cancer progression (Figure 1 and Supplementary Table 1). Our results are thus consistent with a previous study, which determined that Dab2 protein levels were decreased in 74% of breast tumour samples examined (Bagadi *et al*, 2006). To study the role of Dab2 in breast cancer progression, we have utilised two normal mammary epithelial cell lines MCF10A1 and HME5-cdk4. Although the MCF10A1 cell line contains an inactivated p16 locus (Tang *et al*, 2007), overexpression of cdk4 in HME5 cells allows them to overcome p16-mediated growth arrest and continue to grow in culture (Ramirez *et al*, 2003). Breast tissue obtained from normal healthy women was found to contain variant HMECs (vHMECs), which exhibited p16 promoter hypermethylation both *in vitro* and *in vivo* (Crawford *et al*, 2004). When cultured *in vitro*, these vHMECs went on to acquire chromosomal abnormalities seen in premalignant lesions of breast cancer and further progressed to display invasive behaviour, suggesting that p16 inactivation identifies a sub-population of mammary epithelial cells, which possess the potential to develop into malignant lesions (Crawford *et al*, 2004). MCF10A1 and HME5-cdk4 cells thus provide a relevant model system to study the effects of Dab2 downregulation early in breast cancer development.

In our study, we have downregulated Dab2 expression utilising shRNA-mediated technology; however, the mechanism whereby Dab2 expression is decreased in the course of breast cancer development remains undetermined. A recent study detected Dab2 promoter hypermethylation in 11% (6 out of 54) of primary breast tumours, whereas overall 74% (67 out of 91) of breast tumours analyzed displayed decreased Dab2 protein expression, leading the authors to suggest that Dab2 promoter hypermethylation is an infrequent cause for loss of Dab2 expression in breast cancer (Bagadi *et al*, 2006). Although not determined in this study, it is tempting to speculate that Dab2 promoter hypermethylation is associated with a specific subtype of breast cancer, the basal subtype, as these tumours have been shown to specifically exhibit an EMT-like phenotype (Dumont *et al*, 2008; Sarrio *et al*, 2008). Indeed, MDA-MB 231 cells, a metastatic basal-like breast cancer cell line, which expresses EMT markers, exhibits Dab2 promoter hypermethylation (Bagadi *et al*, 2006).

### Regulation of Ras/MAPK signalling by Dab2

TGF*β* treatment has long been known to lead to activation of MAPK signalling; however, until recently the mechanism by which activation of the TGF*β* receptors led to activation of Ras remained unclear. Lee *et al* (2007) reported that activated T*β*RI binds and phosphorylates ShcA proteins on tyrosine and serine residues, which induces the recruitment of Grb2 and Sos to T*β*RI, and hence activation of Ras, Raf and ERK. Here, we show that TGF*β* stimulation leads to increased Grb2/Sos association (Figure 3). As it has already been suggested that a competition exists between Dab2 and Sos for the binding of Grb2 (Xu *et al*, 1998), decreased expression of Dab2 may enhance TGF*β*-stimulated Ras/ERK signalling by shifting the levels of Grb2 available for binding to ShcA. Alternatively, the p96 isoform of Dab2, through its role as an endocytic adaptor molecule (Morris and Cooper, 2001), may direct the TGF*β* receptors to clathrin-coated pits. Athough the TGF*β* receptors have been shown to reside in both clathrin-coated pits and lipid raft compartments of the cell membrane (Di Guglielmo *et al*, 2003), receptor localisation in lipid raft structures was shown to be required for TGF*β*-stimulated MAPK activation and EMT, but not TGF*β*-mediated phosphorylation of Smad2/3 (Zuo and Chen, 2009). Loss of Dab2 expression may thus shift localisation of TGF*β* receptors to lipid raft compartments leading to increased MAPK signalling and EMT.

Cooperation between the Ras/MAPK and TGF*β* signalling pathways has been postulated to be important for induction and maintenance of stable EMT. In mouse EpH4 cells, expression of oncogenic Ha-ras was required for the cells to undergo EMT in the presence of TGF*β* (Janda *et al*, 2002), whereas in human vHMECs, expression of Ha-ras in the presence of 10% serum or TGF*β* was shown to trigger sustained induction of EMT leading to repression of E-cadherin (Dumont *et al*, 2008). Although activation of the Ras pathway is commonly observed in breast cancer, this is not due to oncogenic activation of the Ras proteins (Clark and Der, 1995). We show here that stable downregulation of Dab2 leads to potent stimulation of the Ras/MAPK pathway (Figures 3 and 5), resulting in enhanced expression of TGF*β* and establishment of an autocrine TGF*β* signalling loop, which is consistent with the finding that blockade of MAPK signalling or blockade of TGF*β* signalling decreases the EMT phenotype in Dab2 knockdown cells. Loss of Dab2 expression may thus supply activation of the Ras pathway in breast cancer, which along with other genetic changes, leads to the development of the aggressive and basal subtype of breast cancer.

### Autocrine TGF*β*2 signalling and tumour progression

Although treatment of cells with all three isoforms of TGF*β* has been shown to induce EMT in culture, individual isoforms have been shown to have distinct roles in regulation of EMT in developmental processes. During heart development, it was found that TGF*β*2, but not TGF*β*1 or TGF*β*3, mediated cardiac cushion EMT, which is important for cardiac valve formation (Azhar *et al*, 2009). Transforming growth factor *β*3, on the other hand, is required for induction of EMT in medial-edge epithelial cells, important for palate cleft fusion (Nawshad *et al*, 2007). In breast cancer, overexpression of TGF*β*1 was found to be associated with a higher occurrence of distant metastasis (Tan *et al*, 2009). We find that decreased expression of Dab2 in two normal mammary epithelial cell lines leads to upregulation of the TGF*β*2 isoform, whereas expression of TGF*β*1 and TGF*β*3 isoforms are also stimulated to a lesser extent (Figures 3 and 5). In breast cancer, presence of a novel 4 bp insertion in the TGF*β*2 promoter, which leads to increased promoter activity, was shown to be associated with lymph node metastasis (Beisner *et al*, 2006), suggesting that the TGF*β*2 isoform may contribute to breast cancer metastasis through induction of EMT in mammary epithelial cells.

In conclusion, we show here that downregulation of Dab2, which is commonly observed in breast cancer, can lead to enhanced Ras/MAPK signalling, increased expression of TGF*β*2 and constitutive EMT (Figure 6). Breast cancer metastasis has been correlated with the appearance of an EMT-like phenotype, which is postulated to be controlled by MAPK and TGF*β* signalling. As global targeting of TGF*β* signalling may not be desirable because of its function as a tumour suppressor in normal epithelial cells, strategies targeting TGF*β*2 isoform expression may provide the specificity required to block only the pro-metastatic functions of the TGF*β* signalling pathway. A further understanding of pathways modulated by Dab2, as well as the control of Dab2 expression itself, may thus provide additional targets for intervention in the metastatic process.
