# Supplementary material for: Disabled-2 downregulation promotes epithelial-to-mesenchymal transition
Source: Br J Cancer. 2010 Nov 9;103(11):1716–23. doi: 10.1038/sj.bjc.6605975 (PMC2994233; doi:10.1038/sj.bjc.6605975)
Supplement: Supplementary Data [file 6605975x1.doc]

**Supplemental Data:**

Table 1: Histopathologic data of breast cancer samples

Table 2: Oligonucleotide primers used for qPCR analysis

Supplemental Data Methods

Figure Legends

Figure 1: Decreased Dab2 leads to morphologic appearance of EMT

Figure 2: Growth characteristics of Dab2 knock-down cells

Figure 3: Increased mesenchymal gene expression in Dab2 knock-down cells is maintained in both sparse and confluent cell culture conditions

**Table 1.** Histopathologic Data of Breast Cancer Samples^#^

| Sample | Tumor Type | Grade | ER/PR | ER value  (fmol/mg) | PR value  (fmol/mg) | p96/p67@ |
| --- | --- | --- | --- | --- | --- | --- |
| 1 | Invasive mixed tubular | 5 | 1+/3+ | 7 | 233 | +/ - |
| 2 | Invasive ductal | 9 | 1+/3+ | 14 | 99 | +/ - |
| 3 | Invasive ductal | 6 | 5+/5+ | 142 | 528 | - /- |
| 4 | Invasive ductal | 7 | 2+/- | 20 | 9 | +/ - |
| 5 | Invasive ductal | ND* | 2+/- | 18 | 7 | +/ - |
| 6 | Invasive ductal | 6 | 3+/1+ | 65 | 30 | - / - |
| 7 | Invasive ductal | 5 | 2+/1+ | 30 | 32 | +/ - |
| 8 | Invasive ductal | 6 | 1+/- | 9 | 0.5 | +/ - |
| 9 | Adenoid cystic | - | 2+/1+ | 22 | 14 | +/ - |
| 10 | Invasive ductal | 5 | -/- | 3 | 0 | - / - |
| 11 | Ductal carcinoma in-situ | - | 1+/-/+ | 19 | 13 | +/ + |
| 12 | Invasive ductal | 8 | 1+/1+ | 6 | 26 | - / - |

# Information provided by the manufacturer

* Not determined

@ Determined experimentally from PCR; + designates presence; - designates absence

**Table 2.** Oligonucleotide primers used for qPCR analysis

| **Gene Name** | **Forward Primer 5’→3’** | **Reverse Primer 5’→3’** |
| --- | --- | --- |
| Actin, beta | TCACCCACACTGTGCCCATCTACGA | CAGCGGAACCGCTCATTGCCAATGG |
| Disabled-2 | CTAGCTATTGCAAATGAGGGAAG | GGTAATACTACTTGAACCCAGGAGCA |
| E-Cadherin | TGCCCAGAAAATGAAAAAGG | GTGTATGTGGCAATGCGTTC |
| N-Cadherin | ACAGTGGCCACCTACAAAGG | CCGAGATGGGGTTGATAATG |
| PAI-1 | TCTGCAGACCTGGTTCCCAC | AGCCCCGTAGTTCCATCCTG |
| TGFβ1 | TGAACCGGCCTTTCCTGCTTCTCATG | GCGGAAGTCAATGTACAGCTGCCGC |
| TGFβ2 | TACTACGCCAAGGAGGTTTACAAA | TTGTTCAGGCACTCTGGCTTT |
| TGFβ3 | CTGGATTGTGGTTCCATGCA | TCCCCGAATGCCTCACAT |
| Vimentin | GAGAACTTTGCCGTTGAAGC | GCTTCCTGTAGGTGGCAATC |

**Supplemental Materials and Methods**

*Cell proliferation-MTT assay*

Cells were plated in 24-well plates at a cell density of 2x10^4^ cells per well. The following day, MTT reagent (3-[dimethylthiazol-2-yl]-2,5-diphenyl tetrazolium bromide, 5mg/mL, Sigma Aldrich) was added to each well (1/10 of volume). After 2 hrs, media was removed and formazan crystals were solubilized in 300 μL of DMSO. Absorbance of the resulting solution was determined at 570nM, with background reference determination at 690nM. This assay was repeated for 3 more consecutive days. Following background subtraction, absorbance readings were averaged and expressed relative to readings at day 1, which was set to 1.0. Shown is the mean +/- std dev of assays performed in quadruplicate.

*Immunofluorescence*

Cells were plated in 12-well plates on sterile glass coverslips at a density of 1x10^5^ cells per well. Selected wells were treated with 2.5ng/ml of TGFβ and incubated for 48 hours followed by fixation using 4% paraformaldehyde. Cells were permeabilized in blocking buffer (PBS containing 3% BSA and 0.2% Triton X-100), incubated with primary E-cadherin, Smad2 (51-1300, Zymed) or vimentin antibodies (diluted 1:500) in blocking buffer overnight at 4^o^C, followed by incubation with goat anti-mouse AlexaFluor 594 or anti-rabbit AlexaFluor 488 (Molecular Probes) for 1 hr at 37^o^C. Following extensive washing, coverslips were mounted on glass slides using Vectashield Mounting medium containing DAPI (Vector Laboratories) to visualize cell nuclei. Immunoflourescence was detected at a magnification of 40X using a Leica HB0100 flourescence microscope and images collected with SPOT camera and imaging programs (Diagnostic Instruments). Images for light microscopy were obtained prior to fixation/permeabilization utilizing the same microscope and imaging programs.

**Figure Legends**

**Figure 1** Decreased Dab2 leads to morphologic appearance of EMT. Cells were untreated or treated with 2.5 ng/mL TGFβ for 48 hrs after which time cells were examined by light microscopy (phase) or were processed for immunoflourescence detection of E-cadherin (E-cad, Transduction Labs) and vimentin (Santa Cruz) expression as described in Supplemental Materials and Methods.

**Figure 2** Parental and Dab2 knock-down cells display similar growth kinetics. Cell proliferation in M1 Neg, M1 599 and M1 1059 cells was assessed by MTT assay as described in Supplemental Materials and Methods. Relative cell number was derived from absorbance readings which were normalized to readings obtained on day 1, which were set to 1.0. Shown is the mean +/- std dev of assays performed in quadruplicate for each time point.

**Figure 3** Increased expression of mesenchymal marker proteins does not depend on cell density. M1 Neg, M1 599 and M1 1059 cells were examined for expression of Dab2, N-cadherin and vimentin by qRT-PCR analysis in sparse culture conditions (10-30% confluence) and confluent cell conditions (90-100% confluence) as described in Materials and Methods. Quantitation is expressed relative to levels in M1Neg cells, which is set to 1. Shown is the mean expression value -/+ std dev from an individual experiment; n = 2 independent experiments.

**Supplemental Data Figure 1**

**Supplemental Data Figure 2**

**Supplemental Data Figure 3**
